# Mitochondrial OXPHOS influences immune cell fate: lessons from hematopoietic AIF-deficient and NDUFS4-deficient mouse models

**DOI:** 10.1038/s41419-018-0583-0

**Published:** 2018-05-22

**Authors:** Audrey Bertaux, Lauriane Cabon, Marie-Noëlle Brunelle-Navas, Sandrine Bouchet, Ivan Nemazanyy, Santos A. Susin

**Affiliations:** 1 Cell Death and Drug Resistance in Lymphoproliferative Disorders Team, Centre de Recherche des Cordeliers, INSERM UMRS 1138, Sorbonne Université, Université Paris Descartes, Sorbonne Paris Cité, 75006 Paris, France; 2 Institut Necker-Enfants Malades (INEM), INSERM U1151, Université Paris Descartes, Sorbonne Paris Cité, 75015 Paris, France

Hematopoietic cells can be stimulated to differentiate, proliferate, or die; in each of these contexts, mitochondrial oxidative phosphorylation (OXPHOS) has a critical role. As such, mutations in OXPHOS-related genes are frequently implicated in human mitochondrial diseases^1^. The regulation of OXPHOS and the mitochondrial production of reactive oxygen species (ROS) are also essential for the maintenance of a balance between quiescent and cycling hematopoietic stem cells (HSCs) in bone marrow (BM) and for thymocyte development^2–4^. During OXPHOS, electrons are transferred through a branched chain of multi-protein complexes (complexes I–IV) towards the ATP synthase (complex V). This electron transfer generates up to 36 molecules of ATP per glucose molecule. Mitochondrial ROS are generated from 0.1 to 2% of electrons that escape from the electronic transfer chain (ETC). Alterations in the structure of individual complexes (e.g., complex I) can disorganize the ETC, reduce mitochondrial bioenergetics, and lead to uncontrolled ROS generation^5^.

As well as being a key factor in caspase-independent cell death^6–9^, the mitochondrial protein apoptosis-inducing factor (AIF) is important for functional OXPHOS^10, 11^. We recently reported on the generation of a new AIF knock-out (KO) mouse model in which the protein was specifically ablated in hematopoietic cells (*Vav-1 Cre*^*+*^ *AIF*^*fl/Y*^)^12^. The loss of AIF resulted in a major reduction in ETC complex I, III, and IV proteins, which led to OXPHOS dysfunction, elevated ROS generation, and low ATP production capacity^12^. In turn, these alterations produced pleiotropic hematopoietic defects, including progressive pancytopenia, BM aplasia, changes in the quiescence/proliferation ratios of HSCs and progenitors, alterations in the development of the B-cell and erythroid lineages, and T-cell developmental blockade at the CD4^−^/CD8^−^ double-negative stage. Our study of the AIF KO mouse also revealed that when OXPHOS was significantly impaired, BM cells and thymocytes differed in their metabolic response: the BM cells shifted their metabolism towards anaerobic glycolysis, whereas thymocytes favored fatty acid β-oxidation (FAO). However, this adaptive metabolic response did not prevent the death of the AIF KO mice around 28 days after birth^12^.

**Fig. 1:**
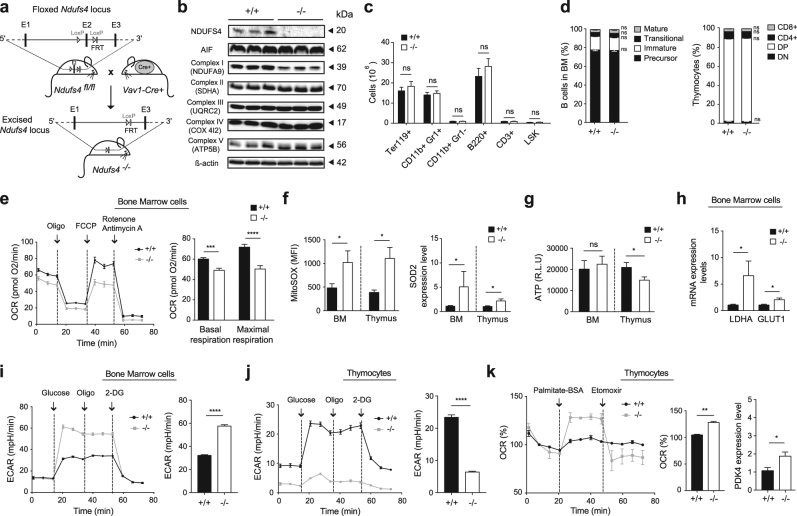
Deletion of NDUFS4 specifically in hematopoietic cells alters mitochondrial OXPHOS and prompts an adaptive metabolic response by BM cells and thymocytes. **a** Crossing *Ndufs4* ^*fl/fl*^ mice^13^ with the *Vav1-Cre*^*+*^ strain induced the excision of Exon 2 (E2) in *Ndufs4* and the creation of a frame shift that prevented the generation of NDUFS4 in hematopoietic cells. **b** A representative immunoblot of BM cells obtained from *Ndufs4*^*+/+*^ (+/+) and *Ndufs4*^*−/−*^ (−/−) animals revealing NDUFS4, AIF, and key ETC complex proteins. Equal cell loading levels were confirmed by probing for β-actin (from *n* = 3 independent experiments, with similar results). **c** Numbers of BM erythroid cells (Ter119+), granulocytes (CD11b+Gr1+), macrophages/monocytes (CD11b+Gr1-), B-cells (B220+), T-cells (CD3+) and Lin^-^Sca-1^+^cKit^+^ (LSK) cells from *Ndufs4*^*+/+*^ (+/+) and *Ndufs4*^*−/−*^ (−/−) animals, measured using flow cytometry (*n* = 8 mice per group). **d** *Left*, cytofluorometric analysis of precursor (IgM-IgD-), immature (IgM+IgD-), transitional (IgM+IgDint), and mature (IgM+IgD+) B220+B-cells from *Ndufs4*^*+/+*^ (+/+) and *Ndufs4*^*−/−*^ (−/−) animals (*n* = 8 mice per group). *Right*, proportions of CD4−/CD8− (double-negative, DN), CD4+/CD8+ (double-positive, DP), CD4+, and CD8+ thymocytes from *Ndufs4*^*+/+*^ (+/+) and *Ndufs4*^*−/−*^ (−/−) animals, measured by flow cytometry (*n* = 8 mice per group). **e** *Left*, a Seahorse oxygen consumption rate (OCR) assay of BM cells from *Ndufs4*^*+/+*^ (+/+) and *Ndufs4*^*−/−*^ (−/−) mice under basal conditions (initial rates) and in response to sequential treatment with oligomycin (an ATP synthase inhibitor; 1 µM), carbonilcyanide p-triflouromethoxyphenylhydrazone (FCCP, an uncoupling agent that enables measurement of the maximum respiration capacity; 1 µM), and rotenone/antimycin A (ETC inhibitors; 1 µM). Arrows indicate the time points at which each reagent was added. *Right*, basal and maximum OCRs of BM cells (shown as histograms, from *n* = 3 independent experiments). **f** Mitochondrial ROS levels in BM cells and thymocytes from *Ndufs4*^*+/+*^ (+/+) and *Ndufs4*^*−/−*^ (−/−) animals were recorded using flow cytometry and the specific MitoSOX probe (1 µM). SOD-2 mRNA levels in BM cells and thymocytes from *Ndufs4*^*+/+*^ (+/+) and *Ndufs4*^*−/−*^ animals were recorded by qPCR using TaqMan® Gene Expression Assays. Data were analyzed using the comparative threshold cycle method. *18S* expression was used to normalize the data (*n* = 6 mice per group). **g** ATP levels in BM cells and thymocytes from *Ndufs4*^*+/+*^ (+/+) and *Ndufs4*^*−/−*^ (−/−) animals were recorded with a bioluminescence assay kit (*n* = 6 mice per group). **h** LDHA and GLUT1 mRNA levels were measured and analyzed by qPCR (as in **f**) in BM cells from *Ndufs4*^*+/+*^ (+/+) and *Ndufs4*^*−/−*^ (−/−) animals. *18S* expression was used to normalize the data (*n* = 6 mice per group). **i** *Left*, the extracellular acidification rate (ECAR) measured using a Seahorse assay in BM cells from *Ndufs4*^*+/+*^ (+/+) and *Ndufs4*^*−/−*^ (−/−) mice, in response to sequential treatment with glucose (10 mM), oligomycin (1 µM), and 2-deoxyglucose (2-DG, an inhibitor of glycolysis; 500 mM). Arrows indicate the time points at which each reagent was added. *Right*, the ECAR of BM cells after glucose treatment (shown as a histogram, from *n* = 3 independent experiments). **j** *Left*, the ECAR of thymocytes from *Ndufs4*^*+/+*^ (+/+) and *Ndufs4*^*−/−*^ (−/−) mice measured using a Seahorse assay as in **i**. Arrows indicate the time points at which each reagent was added. *Right*, the ECAR of thymocytes after glucose treatment (shown as a histogram, from *n* = 3 independent experiments). **k** *Left*, the OCR of thymocytes from *Ndufs4*^*+/+*^ (+/+) and *Ndufs4*^*−/−*^ (−/−) mice in response to the sequential addition of bovine serum albumin-palmitate (BSA-palmitate, a substrate for FAO; 17 µM) and the specific inhibitor of FAO etomoxir (200 µM), measured using a Seahorse assay. Arrows indicate the time points at which each reagent was added. *Middle*, the OCR of thymocytes after BSA-palmitate treatment was expressed as a histogram (*n* = 3 independent experiments). *Right*, PDK4 mRNA expression levels were recorded and analyzed by qPCR (as in **f**) in thymocytes from *Ndufs4*^*+/+*^ (+/+) and *Ndufs4*^*−/−*^ (−/−) mice. *18S* expression was used to normalize the data (*n* = 6 mice per group). Results for samples obtained from *Ndufs4*^*+/+*^ (+/+) and *Ndufs4*^*−/−*^ (−/−) 4-weeks-old animals fed a standard diet (expressed as the mean ± standard error) were compared in a Mann Whitney test. The threshold for statistically significance is indicated as follows: *p ≤ 0.05, ***p ≤ 0.001, and ****p ≤ 0.0001. The materials and methods used (including antibodies and reagents) are similar to those described previously^12^

To better characterize the influence of mitochondrial OXPHOS/metabolism during hematopoiesis, we generated a hematopoietic NADH:ubiquinone oxidoreductase iron-sulfur protein 4 (NDUFS4)-KO mouse by crossing the *Ndufs4* floxed mice^13^ with the *Vav1-Cre*^*+*^ strain^14^ (Fig. 1a). Here, the ETC was less disorganized than in the AIF KO mouse (Fig. 1b). Although the loss of NDUFS4 modified the assembly of mitochondrial complex I, OXPHOS function was retained. This might be due to the stability of complexes II, III, IV, and V, and thus preservation of the ETC’s activity^15^. Consequently, the hematopoietic NDUFS4 KO mice were viable (unlike AIF KO mice^12^) and do not show relevant phenotypic alterations in lymphoid organs (BM, thymus, etc.). There were no significant differences between wild-type (WT, *Ndufs4*^*+/+*^) and hematopoietic *Ndufs4*^*−/−*^ animals with regard to peripheral blood white cell, red cell and platelet counts and erythroid, macrophage/monocyte, B-lymphoid, T-lymphoid and Lin-Sca1 + c-kit + (LSK) BM cell populations (Fig. 1c). B-cell and T-cell development also appeared to be similar in the WT vs. KO animals (Fig. 1d). The *Ndufs4*^*−/−*^ BM cells nevertheless displayed a lower respiratory capacity (Fig. 1e), greater generation of mitochondrial ROS, and higher mRNA expression levels of superoxide dismutase-2 (SOD-2) (Fig. 1f). A similar OXPHOS profile was seen in *Ndufs4*^*−/−*^ thymocytes. Surprisingly, the ATP levels measured in both cell types were very similar to those assessed in WT cells (Fig. 1g) suggesting that the moderate alteration in OXPHOS associated with NDUFS4 loss was either irrelevant for energy generation or was counterbalanced by a metabolic shift (e.g., as seen in AIF-deficient cells)^12^. Quantitative PCR assays of genetic markers of glycolytic activity (*Ldha* and *Glut1*) and measurements of the extracellular acidification rate (ECAR) further indicated that the loss of NDUFS4 in BM cells was compensated by a reinforcement of anaerobic glycolysis (Fig. 1h, i). In *Ndufs4*^*−/−*^ thymocytes, the OXPHOS defects appeared to be counterbalanced by a shift towards FAO, as revealed by a low ECAR rate, a high level of palmitate assimilation, and overexpression of the FAO-facilitating enzyme PDK4 (Fig. 1j, k).

One important lesson from our study of hematopoietic cells in NDUFS4 and AIF KO mice is that regardless of the level of OXPHOS impairment, BM cells quickly adapt their metabolism towards anaerobic glycolysis, whereas thymocytes favor FAO (which requires the maintenance of a mitochondrial OXPHOS activity). It is not clear why the BM cells’ metabolism is directed towards anaerobic glycolysis rather than FAO. One possible explanation is that BM cells decrease the use of mitochondrial pathways (and thus the generation of harmful ROS) as much as possible. It could also be because glucose is more readily available than fatty acids in the BM environment. Furthermore, FAO has to be avoided because it appears to be toxic for BM cells^12^. In contrast to BM cells, thymocytes activate FAO rather than the anaerobic glycolytic pathway. This might be due to the high-energy requirements for thymocyte maturation, selection, and differentiation, and the fact that FAO is a more efficient energy-generating pathway. It is also possible that thymocytes cannot afford to lose TCA cycle intermediates used in other essential biochemical pathways. So, thymocytes may have no choice but to make OXPHOS operate at any cost.

A second lesson concerns the hematopoietic cells’ reaction to mitochondrial ROS. A moderate increase in ROS levels (such as that observed in *Ndufs4*^*−/−*^ cells) provoked similar adaptive responses in BM cells and thymocytes (as judged by mRNA overexpression of the antioxidant SOD-2). Together with the metabolic shift towards anaerobic glycolysis or FAO, this response is enough to restrict the harmful effects of ROS. When the levels of mitochondrial ROS exceed the sustainable limit (after the loss of AIF), the cell keeps trying to regulate ROS levels by increasing the activity of its antioxidant systems^12^. However, the mitochondrial ROS produced by disorganization of the ETC appears to be particularly toxic for thymocytes^12^. Thus, the hematopoietic AIF KO model reveals the ROS “point of no return” for BM cells and thymocytes, and emphasizes the thymocytes’ fragility when exposed to mitochondrial ROS.

Lastly, the hematopoietic NDUFS4 and AIF KO models highlighted the ways that thymocytes generated energy. *Ndufs4*^*−/−*^ thymocytes differentiated normally (e.g., CD4^+^/CD8^+^ cells) and maintained ATP levels in animals fed a standard (carbon) diet. Thus, following the FAO adaptive response, thymocytes might combine fatty acid and glucose fuels to generate ATP. In the context of AIF deficiency, thymocytes responded differently. To generate CD4^+^/CD8^+^ cells, it was mandatory to provide AIF KO thymocytes with fatty acids by feeding the animals a high-fat diet. Hence, in contrast to *Ndufs4*^*−/−*^ thymocytes, AIF-deficient thymocytes mainly use FAO to differentiate and to generate energy.

Taken as a whole, our observations of hematopoietic AIF-deficient or NDUFS4-deficient mice (i) highlighted the fine-tuning of mitochondrial OXPHOS in immune cells, (ii) revealed the various metabolic options available to key hematopoietic subsets, (iii) illustrated the energy requirements of BM cells and thymocytes, and (iv) demonstrated that the signals emitted by mitochondria are critical for cellular decision-making. Better knowledge of how hematopoietic cells can modify their metabolic pathways and can control intracellular ROS levels may enable us to manipulate the development and differentiation of these populations. Ultimately, this might lead to new treatment options for diseases in which hematopoietic or immune cell deregulation has an instrumental role.
